# Supplementary material for: A Novel RNA Virus, Macrobrachium rosenbergii Golda Virus (MrGV), Linked to Mass Mortalities of the Larval Giant Freshwater Prawn in Bangladesh
Source: Viruses. 2020 Oct 2;12(10):1120. doi: 10.3390/v12101120 (PMC7601004; doi:10.3390/v12101120)
Supplement: Supplementary file 1 [file viruses-12-01120-s001.pdf]

viruses

MDPI

Supplementary

A Novel RNA Virus, Macrobrachium rosenbergii Golda Virus (MrGV), Linked to Mass Mortalities of the Larval Giant Freshwater Prawn in Bangladesh

Chantelle Hooper [1,*], Partho P. Debnath [2,*], Sukumar Biswas [3] Ronny van Aerle [1,4], Kelly S. Bateman [1,4], Siddhawartha K. Basak [2], Muhammad M. Rahman [2], Chadag V. Mohan [5], H.M. Rakibul Islam [6], Stuart Ross [1], Grant D. Stentiford [1,4], David Currie [3] and David Bass [1,4,7]

[1] International Centre of Excellence for Aquatic Animal Health, Centre for Environment, Fisheries and Aquaculture Sciences (Cefas), Weymouth, Dorset DT4 8UB, UK
[2] WorldFish Bangladesh, Dhaka 1213, Bangladesh
[3] Winrock Bangladesh, Dhaka, 1212, Bangladesh
[4] Centre for Sustainable Aquaculture Futures, University of Exeter, Stocker Road, Exeter EX4 4QY, UK
[5] WorldFish, Penang, 11960, Malaysia
[6] Bangladesh Fisheries Research Institute, Shrimp Research Station, Bagerhat, Bangladesh
[7] Department of Life Sciences, the Natural History Museum, London, SW7 5BD, UK
* Correspondence: chantelle.hooper@cefas.co.uk (C.H.); p.debnath@cgiar.org (P.P.D.)

Abstract: Mass mortalities of the larval stage of the giant freshwater prawn, *Macrobrachium rosenbergii*, have been occurring in Bangladesh since 2011. Mortalities can reach 100% and have resulted in an 80% decline in the number of hatcheries actively producing *M. rosenbergii*. To investigate a causative agent for the mortalities, a disease challenge was carried out using infected material from a hatchery experiencing mortalities. Moribund larvae from the challenge were prepared for metatranscriptomic sequencing. *De novo* virus assembly revealed a 29 kb single-stranded positive-sense RNA virus with similarities in key protein motif sequences to yellow head virus (YHV), an RNA virus that causes mass mortalities in marine shrimp aquaculture, and other viruses in the *Nidovirales* order. Primers were designed against the novel virus and used to screen cDNA from larvae sampled from hatcheries in the South of Bangladesh from two consecutive years. Larvae from all hatcheries screened from both years were positive by PCR for the novel virus, including larvae from a hatchery that at the point of sampling appeared healthy, but later experienced mortalities. These screens suggest that the virus is widespread in *M. rosenbergii* hatchery culture in southern Bangladesh, and that early detection of the virus can be achieved by PCR. The hypothesised protein motifs of Macrobrachium rosenbergii golda virus (MrGV) suggest that it is likely to be a new species within the *Nidovirales* order. Biosecurity measures should be taken in order to mitigate global spread through the movement of post-larvae within and between countries, which has previously been linked to other virus outbreaks in crustacean aquaculture.

Keywords: *Macrobrachium rosenbergii*; freshwater prawns; *Nidovirales*; Macrobrachium rosenbergii Golda virus; RNA virus; Roniviridae; emerging disease; aquaculture

Table S1. Illumina MiSeq sequencing and assembly statistics.

	**Raw Reads**		**rnaSPAdes Assembly**						**IVA Assembly**					**Read Mapping**	
**Library**	**Paired reads (bp)**	**N50**	**Number of contigs assembled**	**Minimum size (nt)**	**Maximum size (nt)**	**Average size (nt)**	**Contigs mapped to MrGV genome**	**Average Coverage (x)**	**Number of contigs assembled**	**Minimum size (nt)**	**Maximum size (nt)**	**Average size (nt)**	**Contigs mapped to MrGV genome**	**Paired reads mapped to MrGV genome**	**Average Coverage (x)**
1	3,453,843	133	4,713	139	11,163	643.3	11	0.2548	2	1,076	1,146	1,111	0	1,226	5.37
2	3,545,471	155	8,170	132	9,515	696	32	2.5333	20	869	3,393	1,727	0	49,594	269.28
3	5,867,622	209	19,533	131	14,156	674	50	5.04	14	937	14,858	4,457	2	248,277	1462.64
1 + 2 + 3	12,866,936	176	38,826	129	19,260	680	81	7.2555	13	735	29,110	5,046	1	299,141	1629.76

Table S2. Accession numbers and names of nidoviruses used for phylogenetic analyses.

Taxonomic Classification	Species	Acronym	Accession number
Arteriviridae	Equine arteritis virus	EAV	X53459.3
Arteriviridae	Kibale red-tailed guenon virus 1	KRTGV	JX473849.1
Torovirinae	Ball python nidovirus 1	BPNV	KJ541759.1
Torovirinae	Bovine torovirus	BRV	AY427798.1
Mesoniviridae	Mesonivirus 2	MenoV	JQ957873.1
Mesoniviridae	Alphamesonivirus 1	NDiV	DQ458789.2
Mesoniviridae	Turrinivirus-1	-	KX883629.1
Mononiviridae	Planarian secretory cell nidovirus	PSCNV	MH933735.1
Coronavirinae	Rousettus bat coronavirus HKU9	Ro-BatCoV_HKU9	EF065513.1
Coronavirinae	Severe acute respiratory syndrome-related coronavirus	SARS-CoV	AY274119.3
Coronavirinae	Severe acute respiratory syndrome coronavirus 2	SARS-CoV-2	NC_045512.2
Euroniviridae	Charybnivirus-1	-	KX883628.1
Euroniviridae	Decronivirus-1	-	KX883636.1
Euroniviridae	Paguronivirus-1	-	KX883627.1
Roniviridae	Gill-associated virus	GAV	AF227196.2
Roniviridae	Yellow head virus	YHV	EU487200.1
Astroviridae	Chicken astrovirus	CAstV	MK746105.1
Astroviridae	Canine astrovirus	CaAstV	NC_026814.1
